# Future Directions in Choline: From Neurodevelopment to Cardiometabolic Health

**DOI:** 10.3390/nu17223618

**Published:** 2025-11-20

**Authors:** Evan M. Paules, Hannah G. Petry, Jessica K. Sprinkles, Isis Trujillo-Gonzalez

**Affiliations:** 1 Nutrition Research Institute, University of North Carolina at Chapel Hill, Kannapolis, NC 28081, USA; paulese@unc.edu (E.M.P.); hannah_petry@unc.edu (H.G.P.); jsprinks@unc.edu (J.K.S.); 2 Department of Nutrition, Gillings School of Global Public Health, University of North Carolina at Chapel Hill, Chapel Hill, NC 27599, USA

**Keywords:** choline, neurodevelopment, cognition, choline intake, metabolic health, betaine, TMAO, DHA, cardiovascular disease, GLP-1, NuSH

## Abstract

Although choline was established as an essential nutrient over three decades ago, critical questions remain about how choline regulates brain, liver, and cardiometabolic health across the lifespan. This Perspective summarizes emerging insights presented at the *Future Directions in Choline Symposium: A Tribute to Steven H. Zeisel*, which outlined three converging areas of research: (i) choline-dependent mechanisms in neurodevelopment and cognition, (ii) the link between choline metabolism and obesity, and (iii) the role of trimethylamine N-oxide (TMAO) in cardiovascular disease. Evidence from clinical and preclinical studies confirms that maternal choline intake is critical for neurogenesis, cognition, and visual system development, and that higher choline availability buffers the fetal brain against environmental and psychosocial stressors. Choline supplementation improves cognitive outcomes in fetal alcohol spectrum disorder and mitigates neurodegenerative pathology in Alzheimer’s models. In cardiometabolic health, recent data challenge the interpretation of TMAO as a causal toxin, positioning it instead as a marker of renal function. Moving forward, the field must develop validated biomarkers of choline adequacy in free-living populations, harmonize intervention protocols, and define context-specific requirements across obesity, pregnancy, and glucagon-like peptide-1 (GLP-1)-based therapy use. These efforts will refine dietary recommendations and solidify choline’s role in lifelong brain and metabolic health.

## 1. Introduction

More than three decades after choline was defined as an essential nutrient, fundamental questions remain about how choline availability regulates cognition across the lifespan, its role in liver health in obesity, and cardiometabolic health. At the *Future Directions in Choline Symposium: A Tribute to Steven H. Zeisel*, investigators delineated three converging research fronts: (i) choline-dependent mechanisms in brain health, (ii) the link between choline metabolism and obesity and (iii) the contribution of TMAO to cardiovascular risk.

This meeting aimed to evaluate the current state of choline science across the lifespan, from prenatal development through aging, identifying key knowledge gaps in choline biology, and define research priorities that can advance our understanding of its role in human health. Yet, the field now faces the dual challenge of replicating key findings to reinforce mechanistic validity and harmonizing preclinical and clinical approaches to enhance translation. A critical barrier remains the absence of a validated biomarker of choline adequacy in free-living populations where dietary intake cannot be precisely controlled, as current estimates based on food frequency questionnaires and dietary recalls lack precision.

This symposium also honored the career and legacy of Dr. Steven H. Zeisel, Kenan Distinguished University Professor of Nutrition and Pediatrics at the University of North Carolina at Chapel Hill (UNC) and Professor Emeritus in its Department of Nutrition. Dr. Zeisel earned his MD from Harvard Medical School (1975), followed by a pediatric residency at Yale–New Haven Hospital (1975–77). He went on to complete his PhD in Nutrition at the Massachusetts Institute of Technology (1977–80), followed by a post-doctoral fellowship in neurochemistry (1980–81). Dr. Zeisel’s laboratory achieved international recognition for pioneering work on the essential nutrient choline, elucidating its critical roles in neurodevelopment, liver and muscle function, and revealing that individual genetic variation substantially influences choline requirements, a paradigm-shifting discovery for precision nutrition. Under his leadership the Nutrition Research Institute at the North Carolina Research Campus was established to focus on nutrigenomics, metabolomics and metabolic heterogeneity, marking a shift from “one-size-fits-all” dietary guidance to individualized nutrient recommendations. This symposium was held at the North Carolina Research Campus in Kannapolis, North Carolina, and was hosted by the UNC Nutrition Research Institute.

An additional goal of the meeting was to foster alignment among academic, government and industry leaders on evidence-based messages regarding the importance of dietary choline in health promotion and disease prevention.

### Brief Framework of Choline Metabolism

Choline was recognized as an essential nutrient for humans in 1998 by the National Academy of Medicine. Adequate intakes (AI) were established at 550 mg/day for adult men, 425 for adult women, 450 mg/day for pregnant women, and 550 mg/day during lactation [1]. These AIs were derived from experimental evidence by Zeisel et al., who provided the first controlled demonstration that consuming a choline-deficient diet for three weeks led to a 30% reduction in plasma choline and a significant rise in serum alanine aminotransferase (ALT) activity, indicating liver injury. Restoring choline intake reversed these abnormalities, confirming that insufficient dietary choline caused the hepatic dysfunction [2].

**Figure 1:**
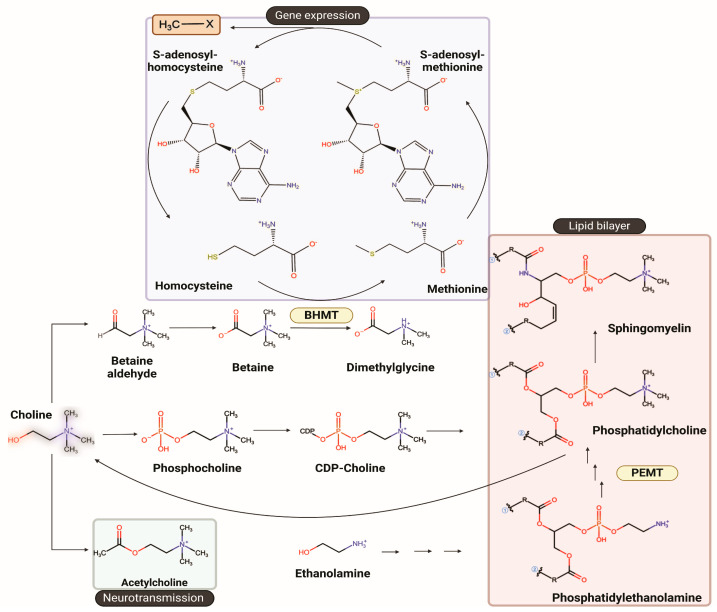
Overview of choline metabolism and its major biochemical pathways. Choline serves as a precursor for metabolites that support neurotransmission, methylation, and cellular membrane synthesis. Through oxidation to betaine, choline donates methyl groups for remethylation of homocysteine to methionine via betaine homocysteine methyltransferase (BHMT), sustaining S-adenosylmethionine-dependent methylation reactions that regulate gene expression. Choline is also phosphorylated and converted through the CDP-choline pathway into phosphatidylcholine, a key structural phospholipid. In parallel, phosphatidylethanolamine N-methyltransferase (PEMT) catalyzes the sequential methylation of phosphatidylethanolamine to phosphatidylcholine in the liver, linking choline metabolism to lipid homeostasis. The structures were drawn using ChemDraw version 25.0.

Choline plays a central role in the synthesis of acetylcholine (Ach), a neurotransmitter integral to cognitive functions, and its impact on learning and memory [3,4,5,6,7]. Choline is the precursor of betaine, which is a methyl donor for the synthesis of the universal methyl donor S-adenosylmethionine (SAM) for methylation reactions such as DNA and histone methylation, that are well-known epigenetic mechanisms involved in the regulation of gene expression. Additionally, choline is crucial for cell membrane formation, myelination and lipid transport (Figure 1). This biochemical framework establishes the basis for understanding how genetic variants that alter choline availability increase dietary choline requirements and, in turn, influence brain function, liver metabolism, and cardiometabolic health.

This Perspective integrates recent advances within these domains and defines the questions that will guide the next phase of choline research.

## 2. Dietary Choline Intake, Neurodevelopment and Cognition

Choline is part of one-carbon metabolism, and genetic variants in the methylenetetrahydrofolate reductase (*MTHFR*) gene increase choline requirements [8]. Moreover, vitamin B12 is a cofactor for methionine synthase, the enzyme that remethylates homocysteine to methionine. Inadequate B12 intake compromises the synthesis of the universal methyl donor SAM, making choline and its metabolite betaine increasingly important in supporting methyl donors that regulate gene expression [9].

Pregnancy represents a critical window for choline availability because of its essential role in maternal liver function, placental integrity, and fetal brain development. Strikingly, women of reproductive age worldwide fail to meet choline intake recommendations [10,11,12]. Given the metabolic intersection between folate and choline, and the well-established 50% reduction in neural tube defect risk with adequate folate intake, Dr. Obeid posed the question of whether choline could confer protection. Direct experimental testing is constrained by feasibility and ethics, considering a clinical trial would require >10,000 participants and cannot ethically withhold folate. Instead, Obeid conducted a rigorous meta-analysis demonstrating that low maternal choline intake or circulating choline concentrations are associated with a 36% higher risk of neural tube defects. Furthermore, controlled trials supplementing 550–1000 mg choline per day during late pregnancy improved visual memory and visuospatial learning without adverse effects. Across 30 human studies, the evidence converges on a clear message: most women do not consume sufficient choline, underscoring the need to include this nutrient routinely in prenatal supplements [13].

Perinatal choline intake appears to have long-lasting effects on offspring cognition. Evidence from a randomized controlled feeding trial showed that women who consumed 930 mg/day of choline during the third trimester of pregnancy, compared with 480 mg/day, had children who demonstrated enhanced cognitive performance at school age (7 years old) [14]. These findings align with extensive preclinical data indicating that choline is particularly critical during gestation [15]. The proposed mechanism involves sustained cholinergic support within the frontal cortex, a region essential for attention and executive function. However, the study included only 20 children, with outcomes derived from a single attention task. Larger, diverse trials are needed to establish the optimal dose and timing of choline supplementation. Despite these limitations, this study provides compelling evidence that maternal choline intake during late pregnancy can shape long-term neurocognitive outcomes, reinforcing the nutrient’s relevance in prenatal nutrition.

These human findings are supported by mechanistic evidence from preclinical models that clarify how maternal choline availability shapes brain development. Major advances have shown that neural progenitor cells require choline during neurogenesis, marking this as a sensitive developmental window. In these models, choline availability regulates microRNAs in addition to histone methylation within neural progenitor cells, influencing whether they undergo self-renewal or differentiation into neurons in the cerebral cortex [16,17,18]. In addition to its effects on brain development, choline is also required for proper retinogenesis. This is not unexpected given that neural progenitor cells and retinal progenitor cells share a common embryonic origin but diverge as development progresses [19]. In a mouse model, low choline in utero disrupted retinal development and later visual function. Under low choline conditions, retinas exhibited structural abnormalities, including displaced neurons, reduced cellularity, and fewer differentiated neurons, while retaining a higher proportion of retinal progenitor cells. The lengthened cell cycle of these progenitors was accompanied by reduced expression of Merlin, an upstream regulator of Hippo signaling. Mice exposed to low choline in utero also showed marked intraindividual variation in vision, demonstrating that early nutritional environment exerts lasting functional effects [20]. As noted above, in humans, one of the most consistent findings among children of mothers with low choline intake is the disruption of visuospatial memory. This raises the possibility that part of the cognitive phenotype could arise from altered visual cortical function secondary to aberrant retinal development, as observed in some models of glaucoma [21].

Klatt et al. introduced an important dimension to the field by testing whether prenatal choline supplementation enhances maternal and fetal docosahexaenoic acid (DHA) status, reframing the discussion toward nutrient–nutrient interactions rather than isolated nutrient effects. Choline and DHA intersect metabolically through the phosphatidylethanolamine N-methyltransferase (PEMT) pathway in the liver, where methyl groups derived from choline drive the synthesis of phosphatidylcholine species, which can be enriched in DHA fatty acyl chains [22]. These phosphatidylcholine species can subsequently be exported to systemic circulation [23]. This raises a key question: does elevated choline intake improve DHA status in the mother and fetus during pregnancy by increasing PEMT activity? In a randomized controlled trial of 30 pregnant women, supplementation with 550 mg of choline and 200 mg DHA per day from 12 to 16 weeks of gestation until delivery significantly increased maternal plasma phosphatidylcholine–DHA and plasma DHA concentrations at delivery in addition to a positive trend on newborn biomarkers of DHA status [24]. Despite its modest scale, this study provides mechanistic human evidence that choline status modulates DHA bioavailability, underscoring the importance of considering interdependent nutrient networks in maternal–fetal metabolism.

Another critical window for brain development is lactation, which provides the nutrients necessary to sustain postnatal growth and maturation. This period is particularly important for the establishment of cholinergic neurotransmission and the myelination process, which begins prenatally and accelerates during the first two years of life [25,26,27,28]. Human milk functions as a dynamic nutrient system, delivering fatty acids, phospholipids, and choline that work together to support cognitive and emotional development. Recent findings specifically link DHA, arachidonic acid, and phosphocholine in human milk with early language and temperament traits, effects that persist through the first 18 months of life [29]. Greater attention is needed to understand how milk composition directly influences neurodevelopmental trajectories, including through the integration of neuroimaging technologies to assess its impact on brain function.

### Choline and Food Insecurity

Choline is not only essential during pregnancy for maternal and fetal health but remains critical for childhood growth and brain development. In the United States, about ~60% of children aged 1–6 years do not achieve their AI for choline [30,31,32,33], a gap that also affects regions facing food insecurity and malnutrition. Improving overall nutrient quality is therefore critical. Building on the growing recognition of DHA’s role in neurodevelopment, Manary et al. investigated whether reformulating ready-to-use therapeutic foods (RUTF) could enhance cognitive recovery in Malawian children with severe acute malnutrition. The addition of DHA to the RUTF formulation significantly improved cognitive outcomes beyond standard treatment, providing the first direct evidence that nutrient composition of therapeutic foods can influence neurocognition [34]. These findings strengthen the case for rethinking RUTF formulations to include not only DHA but also choline, given their shared roles in supporting brain structure and function.

## 3. Choline, Environmental Exposures and Neurodegenerative Risk

### 3.1. Choline and Cannabis Exposure In Utero

Maternal psychosocial stressors and toxic exposures directly affect both maternal and fetal health. Hunter et al. examined how mood, stress, and cannabis use influence early childhood outcomes and how these factors intersect with race and ethnicity. Their study focused on the interaction between maternal plasma choline levels, cannabis exposure, and gestational infection in shaping neurobehavioral development at 4 years of age. Higher maternal choline concentrations at 16 weeks of gestation were associated with fewer attention problems and reduced social withdrawal in children. Notably, elevated choline levels mitigated the adverse effects of maternal infection and cannabis exposure, suggesting that choline functions as a physiological buffer that protects the fetal brain from environmental and toxic stressors [35,36].

A critical but often overlooked aspect of psychosocial stress during pregnancy is its disproportionate impact on Black American women, who experience higher rates of depression and prenatal stress. At 16 weeks of gestation, Black American women exhibited significantly lower plasma choline concentrations than White women (5.48 μM vs. 6.58 μM), a difference associated with a threefold higher incidence of preterm birth and overall shorter gestation. Follow-up of these children revealed greater vulnerability to neurodevelopmental and behavioral problems linked to early choline deficiency [37]. These findings raise a critical question for public health and clinical nutrition: are we missing the opportunity to implement prenatal choline supplementation as a preventive strategy to reduce neuropsychiatric risk?

### 3.2. Choline and Fetal Alcohol Spectrum Disorder

One of the most prevalent yet preventable causes of neurobehavioral impairment is prenatal alcohol exposure. In the United States, an estimated 2–5% of first-grade children meet diagnostic criteria for fetal alcohol spectrum disorder (FASD) [38]. Choline supplementation stands out as one of the few nutritional interventions with proven efficacy for FASD. In a randomized, double-blind, placebo-controlled trial, children with FASD given supplemental choline improved nonverbal intelligence, visuospatial performance, working and verbal memory, and behavioral symptoms of attention deficit hyperactivity disorder, highlighting its potential to partially restore neurocognitive function in this population [39]. Long-term follow-ups and aggregate randomized clinical trials (RCT) data also demonstrated improvements in white matter microstructure, executive function, and imitation memory performance [40,41]. Additionally, genetic polymorphisms in the low-affinity choline transporter, solute carrier family 44 member 1 (SLC44A1), were associated with better cognitive outcomes in children with FASD given supplemental choline relative to those who do not. This underscores the need for more targeted interventions with supplemental choline to confer the best outcomes for individuals with FASD [42].

These robust, long-term RCTs have clearly demonstrated that supplemental choline helps mitigate FASD symptomology. Additional RCTs in other populations will solidify the generalizability of providing supplemental choline as a recommendation for children with FASD. However, significant care must be taken to avoid miscommunicating that adding supplemental dietary choline allows for pregnant women to continue substance use.

### 3.3. Choline and Alzheimer’s Disease

Beyond neurodevelopment, high choline intake supports brain health across the lifespan and is associated with a lower risk of incident dementia and Alzheimer’s Disease (AD) [43,44]. Higher intake of phosphatidylcholine, a major circulating choline metabolite, has likewise been linked to better cognitive performance and reduced dementia risk [45]. Preclinical models of AD show that adequate choline intake preserves metabolic health and slows cognitive decline, while low choline accelerates disease progression [46,47]. Lifelong supplementation in these models also reduces amyloid-β plaque burden and mitigates hallmarks of AD pathology [48]. In addition, dietary choline has been shown to reduce lipid accumulation in human astrocytes, a mechanism hypothesized to contribute to age-related cognitive decline particularly in carriers of *APOE4* genetic variants [49]. Despite these advances, a key gap remains in understanding whether choline’s neuroprotective effects act directly within the brain, indirectly through systemic metabolic improvement, or through both in concert.

## 4. Gaps and Future Directions in Choline Brain Health Across the Lifespan

**Figure 2:**
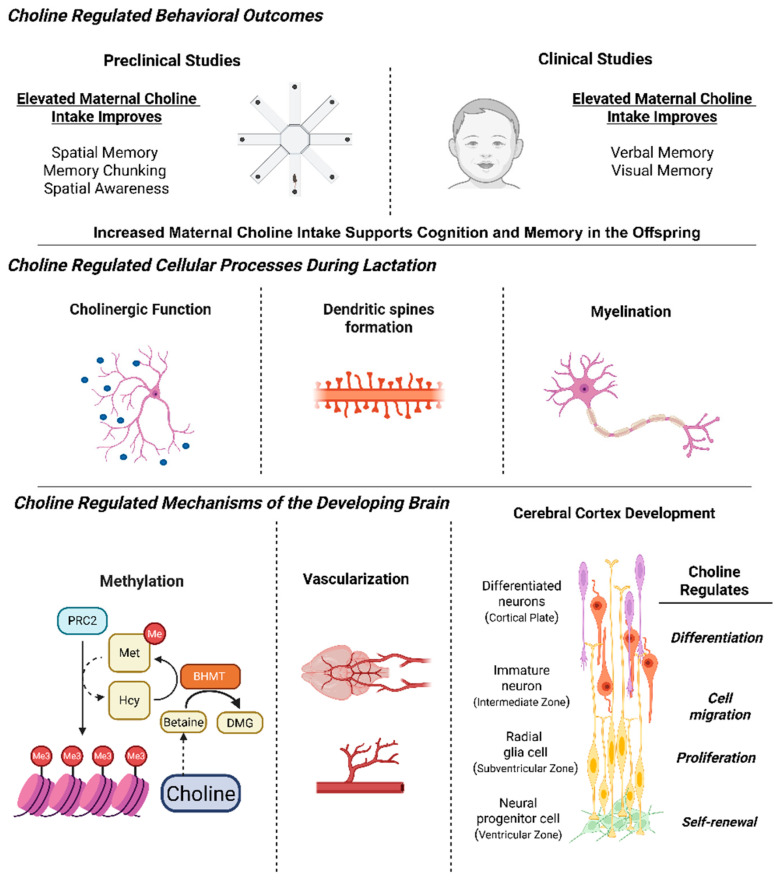
The essential role of choline in brain development and its long-lasting effects in both mice and humans. Preclinical and clinical studies have identified maternal choline intake to impact cognition and memory. During lactation, choline is crucial for cholinergic function, dendritic spine formation and myelination. Mechanistically, choline influences methylation potential, gene expression, vascularization of the brain and the proliferation and differentiation of neural progenitor cells. (Image created with templates from Biorender).

Compelling evidence links dietary choline intake during pregnancy and lactation to offspring cognitive outcomes (Figure 2). However, there is a critical need to harmonize choline intervention protocols during pregnancy. Current trials vary widely in design, with some comparing 930 mg/day to 480 mg/day during the third trimester of pregnancy [7] and others providing 900 mg/day from week 16 of gestation through delivery, followed by 100 mg/day to infants [50,51]. These studies have advanced the field but differ in timing, dosage, and duration, making it difficult to define optimal supplementation windows. Standardized protocols with consistent endpoints and adequate sample sizes are needed to establish evidence-based recommendations for maternal and infant choline intake. In preclinical models, variability in how choline is delivered, through diet, gavage, or intraperitoneal or subcutaneous injections, continues to complicate interpretation of results. The same applies to in vitro systems, where culture conditions and choline concentrations are often inconsistent across studies. Harmonization of these approaches will strengthen the translational bridge between cellular, animal, and human work and clarify how choline interacts with drugs or other nutrients at the mechanistic level.

The influence of the maternal microbiome is another major gap. Differences in choline-metabolizing and trimethylamine-producing bacteria can alter choline bioavailability and its metabolic fate [52], potentially affecting both maternal physiology and fetal development. Understanding these microbial contributions is essential for interpreting interindividual variability in response to choline intake.

Finally, the next phase of choline research must adopt an integrated framework that considers nutrient–nutrient and nutrient–metabolite interactions alongside genetic and metabolic context. Nutrients such as DHA, folate, and vitamin B12 share metabolic pathways with choline, and studying them collectively will provide a more complete picture of how these networks influence development and long-term health. Extending this system’s approach to disease models such as AD is essential to define who benefits most from choline, under which genetic conditions, and at what dosage. The neuroprotective potential of choline is well established, yet progress now depends on coordinated, mechanistic studies that clarify its targets and translational applications.

## 5. Choline and Cardiometabolic Health

### 5.1. Choline and Metabolic Syndrome

Obesity represents a major public health concern, affecting over 42% of adults in the United States [53,54]. This condition increases the risk of metabolic dysfunction-associated steatotic liver disease (MASLD), as well as type 2 diabetes, hypertension, and cancer [55,56]. Higher dietary choline intake has been associated with lower body fat percentage and improved metabolic markers in individuals with metabolic syndrome [57,58]. Moreover, betaine, a key choline-derived metabolite, has been shown in preclinical models to reduce body weight, enhance glucose homeostasis, and increase oxidative capacity in white adipose tissue [57,59]. In humans, lower plasma betaine concentrations have been observed in overweight Mexican Americans, highlighting a potential link between impaired one-carbon metabolism and metabolic dysregulation [60].

Dr. Paules raised an important question regarding whether choline requirements differ between individuals with obesity and those with normal weight. There is a clear need to determine habitual dietary choline intake in obese populations and to elucidate the mechanistic links between choline availability and cardiometabolic outcomes. Recent studies in diversity outbred obese mice have revealed substantial heterogeneity in the metabolic response to calorie restriction, which can be predicted by circulating markers such as leptin. Notably, these metabolic signatures differ between males and females, underscoring the need to incorporate sex-specific analyses when defining choline metabolism and its relationship to metabolic health [61,62]. This question is highly relevant for translational research, as it raises the possibility that current dietary choline recommendations may need to be reconsidered in light of the heterogeneous metabolic responses observed across individuals.

Considerations for dietary choline intake must extend beyond obesity to include the widespread use of nutrient-stimulated hormone (NuSH) therapies, formerly known as incretin mimetics or GLP-1 agonists [63]. While these therapies show great promise in reducing obesity prevalence, they may also alter micronutrient bioavailability and absorption, potentially leading to nutrient deficiencies. This raises critical questions for future research: Do NuSH users develop choline deficiency, and who among them may benefit from targeted choline supplementation?

In parallel, the intersection between choline metabolism and cardiometabolic health is drawing renewed attention. Choline is not only an essential methyl donor but also a substrate for gut microbial metabolism producing trimethylamine (TMA) and its hepatic oxidation product, trimethylamine N-oxide (TMAO). Sprinkles et al. presented findings from a longitudinal, population-based cohort study examining the associations of plasma choline, betaine and TMAO concentrations with 15-year risk of incident diabetes. Using data from the Coronary Artery Risk Development in Young Adults (CARDIA), higher plasma betaine concentrations were strongly and inversely associated with incident diabetes [64]. This observation aligns with mechanistic evidence mentioned above, linking betaine to pathways involved in glucose metabolism and insulin sensitivity. Because plasma betaine reflects, in part dietary choline intake, these findings suggest that higher choline intake may reduce diabetes risk.

### 5.2. TMAO and Cardiovascular Health

TMAO is generated through the hepatic oxidation of TMA by flavin-containing monooxygenase 3 (FMO3). TMA itself originates from the microbial metabolism of choline, phosphatidylcholine, and L-carnitine in the gut [65]. Early metabolomic and epidemiologic studies, such as those from the Framingham Heart Study, associated higher circulating TMAO concentrations with an increased risk of cardiovascular disease (CVD) and chronic kidney disease (CKD) [66]. However, subsequent analyses revealed that these associations are strongly modified by renal function. As kidney function declines, TMAO accumulates, and plasma concentrations in CKD patients can be fivefold higher than in healthy individuals [67]. Mechanistically, TMAO levels rise up to 40-fold above normal in patients receiving chronic dialysis, despite effective removal during treatment [68]. This disproportionate accumulation of TMAO’s high native renal clearance and small volume of distribution makes intermittent dialysis inefficient at maintaining low average concentrations.

Dr. Bortz noted during the discussions that these findings call into question whether TMAO is truly causal in CVD or merely a marker of impaired kidney function. Several studies have reported associations between plasma TMAO concentrations and renal function [69,70], including a recent study showing that plasma TMAO concentrations markedly decrease following kidney transplantation [71]. Other studies have also suggested that associations between TMAO and clinical outcomes may be confounded by kidney function, which could explain the inconsistent findings reported in the epidemiologic literature [72,73]. Mechanistically, TMAO does not appear to promote foam cell formation or cholesterol efflux dysfunction, and its production depends on a minor subset of gut microbes (<0.2% of taxa) possessing TMA-lyase activity.

In a rigorous population-based study, Dr. Katie Meyer examined the relationship between choline metabolism and cardiovascular disease (CVD) risk in younger adults. Using data from the 19-year prospective CARDIA cohort of more than 3400 participants, plasma concentrations of choline, betaine, and TMAO were measured to evaluate their association with incident CVD. The findings challenge the notion of TMAO as a direct causal factor and instead suggest that its predictive value primarily reflects renal clearance capacity rather than a mechanistic role in disease development [74].

Collectively, these data suggest that TMAO is more reflective of renal function than a direct toxin, and that its transient elevation following choline or fish consumption is unlikely to be harmful in individuals with normal kidney function. The challenge ahead is to reconcile epidemiologic associations with mechanistic evidence, refining our understanding of when TMAO serves as a pathological marker versus a benign metabolic readout of one-carbon and renal physiology.

## 6. Gaps and Future Directions in Choline and Cardiometabolic Health

Most epidemiologic research on choline has been conducted in normal-weight populations, limiting our ability to generalize findings to a world in which over 1.5 billion people live with overweight or obesity. This is a critical oversight, as metabolic phenotype, body composition, and inflammation may alter both choline requirements and utilization. It remains unknown whether the current AI values are sufficient for individuals with obesity, who may have altered hepatic metabolism, reduced PEMT activity, or impaired choline absorption. These uncertainties extend to other micronutrients and highlight the need for more precise, context-specific recommendations. The intersection of obesity, pregnancy, and emerging pharmacotherapies introduces additional complexity. Obesity during gestation increases the risk of preeclampsia, gestational diabetes, and altered placental metabolism, all of which may affect maternal–fetal choline transfer and neurodevelopmental outcomes. Moreover, limited studies have also been conducted defining the impact of altered microbiome compositions on TMAO production in metabolic disease states independently of precursor intake. The recent, widespread use of NuSH therapies, such as GLP-1 receptor agonists, adds another layer of uncertainty. These drugs alter nutrient absorption, gastrointestinal motility, and hepatic lipid metabolism, raising important questions about whether they also modify choline status or its metabolic handling.

## 7. Conclusions

The field of choline research continues to evolve, and new findings must be integrated into a more unified framework. There are windows of time when choline demand is particularly high, such as during pregnancy, and evidence supports that targeted supplementation during this period is both beneficial and safe. Circulating choline levels and metabolic requirements in obesity remain unknown. The broad use of novel weight loss NuSH therapies, including GLP-1 receptor agonists, may further compromise choline absorption and metabolism, as observed with other micronutrients, and this warrants careful attention. These gaps highlight the need to reconsider current AI values given the changing characteristics of modern populations and to advance toward a precision nutrition approach in dietary reference value development. A consistent theme across discussions is the urgent need for validated biomarkers of choline status in free-living populations, where dietary intake cannot be precisely controlled. A recent controlled feeding study has shown that plasma choline and betaine concentrations together can discriminate whose dietary choline intake meets the AI [75], yet these relationships require validation in larger and more diverse cohorts. To facilitate the integration of these key findings and to identify remaining gaps, we summarize the major themes discussed across life stages, metabolic contexts, and methodological approaches in Table 1. Addressing these gaps will be essential to translate mechanistic insights into reliable measures of choline adequacy and inform future dietary recommendations across diverse physiological and metabolic contexts.

## Figures and Tables

**Table 1:** Summary of key findings and future directions discussed at the Future Directions in Choline.

Topic	Key Findings and Advances	Translational or Clinical Implications	Identified Research Gaps/Future Directions
**Neurodevelopment and Cognition**	Maternal choline intake during pregnancy supports fetal neurogenesis, attention, and memory; supplementation (550–930 mg/day) improves offspring cognition.	Reinforces inclusion of choline in prenatal supplements; potential long-term cognitive benefits.	Define optimal dose and timing; harmonize intervention protocols; expand trials to diverse populations. Molecular Mechanisms of Cognitive Function
**Nutrient Interactions**	Choline enhances maternal DHA status via PEMT pathway; increased PC–DHA with combined supplementation.	Highlights interdependence of choline and DHA for fetal neurodevelopment.	Clarify nutrient–nutrient interactions and their implications for supplementation policy.
**Environmental and Psychosocial Stressors/FASD**	Higher maternal choline mitigates adverse effects of cannabis exposure, infection, and stress on child attention and behavior. Choline supplementation improves cognition, memory, and white matter microstructure in children with FASD.	Suggests choline acts as a neuroprotective buffer during pregnancy and provides one of the few evidence-based nutritional therapies for FASD.	Identify mechanisms and at-risk populations for targeted interventions.
**Alzheimer’s Disease and Aging**	Adequate choline intake associated with lower dementia risk.	Supports a neuroprotective role for choline across lifespan.	Clarify mechanisms linking choline metabolism, APOE genotype, and glial lipid metabolism.
**Metabolic Health and** **Obesity/NuSh therapies**	Choline and betaine improve body composition and metabolic markers; lower betaine in obesity. GLP-1 receptor agonists may alter nutrient absorption and metabolism.	Suggests altered one-carbon metabolism in obesity. Raises concern for potential choline deficiency in NuSH users.	Reassess Adequate Intake values for individuals with obesity; incorporate sex differences. Determine whether NuSH therapy modifies choline requirements or bioavailability.
**Cardiovascular Health**	TMAO reflects renal clearance rather than direct CVD causality.	Reframes TMAO as a biomarker of kidney function.	Differentiate pathological vs. physiological TMAO elevations; integrate renal function in future analyses.
**Cross-cutting Needs**	Plasma choline and betaine concentrations can serve as predictive markers of dietary choline intake in controlled feeding studies.	Data derived from these biomarkers can support evidence-based revisions of Adequate Intake (AI) values and improve public health nutrition policies.	Validate plasma choline and betaine as biomarkers of dietary intake in free-living populations and determine how disease states alter their predictive value.

## Data Availability

No new data were created or analyzed in this study.

## Abbreviations

The following abbreviations are used in this manuscript:

Ach	acetylcholine
AI	adequate intake
ALT	alanine aminotransferase
AD	Alzheimer’s Disease
CVD	cardiovascular disease
CKD	chronic kidney disease
DHA	docosahexaenoic acid
MASLD	dysfunction-associated steatotic liver disease
FASD	fetal alcohol spectrum disorder
GLP-1	glucagon-like peptide-1
*MTHFR*	methylenetetrahydrofolate reductase
NuSH	nutrient-stimulated hormone therapies
PEMT	phosphatidylethanolamine N-methyltransferase
RCT	randomized clinical trials
RUTF	ready-to-use therapeutic foods
SAM	S-adenosylmethionine
TMA	trimethylamine
TMAO	trimethylamine N-oxide
